# Characterization of Stable Pyrazole Derivatives of Curcumin with Improved Cytotoxicity on Osteosarcoma Cell Lines

**DOI:** 10.3390/life13020431

**Published:** 2023-02-03

**Authors:** Giordana Feriotto, Riccardo Rondanin, Paolo Marchetti, Federico Tagliati, Simone Beninati, Claudio Tabolacci, Elisa Grusi, Serena Aguzzi, Carlo Mischiati

**Affiliations:** 1 Department of Chemical, Pharmaceutical and Agricultural Sciences, University of Ferrara, 44121 Ferrara, Italy; 2 Department of Neuroscience and Rehabilitation, University of Ferrara, 44121 Ferrara, Italy; 3 Department of Biology, University of Rome “Tor Vergata”, 00133 Rome, Italy; 4 Department of Oncology and Molecular Medicine, Istituto Superiore di Sanità, 00161 Rome, Italy

**Keywords:** curcumin, derivatives, apoptosis, pyrazole, osteosarcoma

## Abstract

Curcumin (CUR) is a natural molecule that is unstable due to the presence of a bis-ketone. To obtain more stable derivatives in biological fluids, the bis-ketone was replaced with pyrazole or O-substituted oximes. Their stability in solution was studied by UV–visible spectrophotometry. The effects on proliferation were studied by MTT assay and/or clonogenicity assay. Induction of apoptosis was evaluated by annexin V staining and Western blot analysis. The bioavailability was obtained from the analysis of the molecular chemical–physical characteristics. The replacement of the bis-ketone with a pyrazole ring or O-substituted oximes improved the stability of all the CUR-derivative molecules. These derivatives were more stable than CUR in solution and were generally cytotoxic on a panel of cancer cell lines tested, and they promoted caspase-dependent apoptosis. Derivative **1** was the most potent in the osteosarcoma (OS) lines. With respect to CUR, this derivative showed cytotoxicity at least three times higher in the MTT assay. In addition, in the clonogenic assay, **1** maintained the activity in conditions of long treatment presumably by virtue of its improved stability in biological fluids. Notably, **1** should have improved chemical–physical characteristics of bioavailability with respect to CUR, which should allow for reaching higher blood levels than those observed in the CUR trials. In conclusion, **1** should be considered in future clinical studies on the treatment of OS, either alone or in combination with other medications currently in use.

## 1. Introduction

Osteosarcoma (OS) is a highly malignant neoplasm that is capable of producing metastases mainly in the lung but also in different bone sites. Its tendency to spread is so high that about 80% of patients have micrometastases at the first diagnosis of the disease [1]. A multi-modal therapy, consisting of neoadjuvant preoperative systemic poly-chemotherapy, followed by localized surgery and postoperative adjuvant chemotherapy, resulted in a long-term disease-free survival within 5 years from the treatment in 65–70% of patients with localized OS, but this value decreases to 6% in patients with metastases [2]. On the one hand, these clinical results represent a certain efficacy in OS therapy; and on the other hand, new drugs for the treatment of recurrent or non-responsive forms of OS would be desirable.

Curcumin (CUR) is a polyphenol that is particularly abundant in the rhizome of *Curcuma longa* L., belonging to the Zingiberaceae family. The subsequent solvent extraction and purification by crystallization yield a yellow-orange powder, which is rich in curcumin and has healthy properties. In traditional medicine, the powder was used as a valuable remedy for respiratory diseases and other disorders, such as anorexia, rheumatism, and sinusitis [3]. Growing evidence has demonstrated CUR’s ability to inhibit the proliferation of a wide variety of cancer cells, and numerous clinical trials have tested its potential for both cancer prevention and therapy, as well as in alleviating the side effects of chemotherapy and radiotherapy [4]. CUR inhibits cell proliferation by determining cell cycle arrest in the G2/M phase [5] and modifying the expression and activity of the proteins involved in apoptotic mechanisms [6]. One aspect that made CUR particularly attractive as a possible anticancer therapeutic was its low toxicity profile [7]. In this respect, 75 trials are considering the possible use of CUR in human cancers to date, alone or in combination with current anticancer agents, and 12 are still recruiting patients [8]. Despite this great interest in the molecule, the completed studies highlighted the low bioavailability of CUR. After an oral intake at a high dosage, the CUR level observed in the plasma spanned from 0.4 to 3.6 µM one hour after the administration and then rapidly descended [9]. The low bioavailability was a sum of poor molecular qualities, such as low solubility and low stability in aqueous solution [10], low intestinal absorption, rapid metabolism, and rapid elimination through feces and urine [11,12]. Therefore, CUR-derived analogs and delivery systems have attempted to improve CUR bioavailability [13,14,15,16]. Some of them have been investigated for cytotoxicity and apoptosis-triggering effects in OS tumor cells in vitro and in vivo, but their clinical efficacy is still unknown or unsatisfactory (for a review, see [17]).

The improvement of the stability of CUR in biological fluids represents one aspect to consider for improving its bioavailability. In fact, CUR was quite unstable, and 96% of its molecules undergo degradation to ferulic acid, feruloyl methane, vanillin, and acetone within one hour in physiological pH solutions [18,19,20]. Its instability in a weakly basic pH environment was due to the bis-ketone group [21,22,23]. At a slightly alkaline pH, the C4 was deprotonated and caused the breaking of the bond between C4 and C5, with the formation of ferulic acid and feruloyl methane [19,20]. Therefore, the replacement of the bis-ketone with groups capable of decreasing the acidity of the hydrogen in C4 could be useful to confer ameliorated stability to the CUR derivatives. In this study, we considered the replacement of the bis-ketone with pyrazole or O-substituted oximes in the CUR molecule to obtain more stable derivatives. In this study, we investigated the stability of CUR-derivatives in biological fluids and drug-like characteristics, as well as their cytotoxicity and ability to trigger apoptosis on OS cells.

## 2. Materials and Methods

### 2.1. Preparation of the Curcumin Analogs

**Scheme 1:**
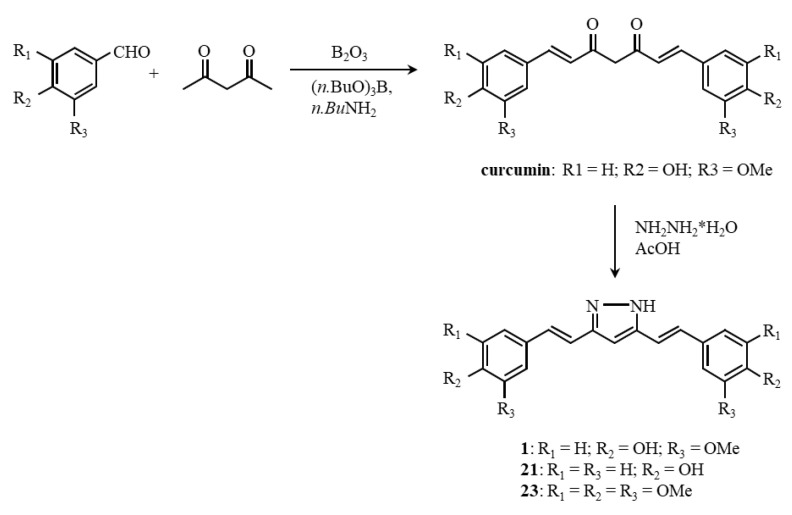
Synthesis of curcumin pyrazole analogs **1**, **21**, and **23**.

Curcumin and its pyrazole derivatives **1**, **21**, and **23** were prepared as depicted in Scheme 1.

Pentan-2,4-dione (10 mmol) and B_2_O_3_ (7 mmol) were dissolved in 10 mL of AcOEt and heated with reflux for 30 min. At the solution, 20 mmol (n.BuO)_3_ and the suitably substituted benzaldehyde (20 mmol) were added. After another 30 min at reflux, n.BuNH_2_ (15 mmol) was added, and the final reaction mixture was stirred overnight at 40 °C. After this time, 30 mL HCl 4N was added, and the mixture was stirred for 30 min at 60 °C. After evaporation, the residue was diluted with 20 mL water and extracted four times with 30 mL AcOEt. Drying of the collected organic phase gave a residue that was purified by column chromatography to give, sequentially, curcumin and curcumin analogs, to which 25 mL glacial AcOH and 10 mmol hydrazine hydrate were added. The solution was refluxed for 8 h, and then the solvent was removed in a vacuum. The residue was dissolved in water and extracted four times with 30 mL CH_2_Cl_2_. The collected organic phase was dried over sodium sulfate and concentrated in a vacuum. The crude mixture was further purified by column chromatography to give pyrazole derivatives **1**, **21**, and **23**. The curcumin O-substituted oximes **3**, **4**, and **5**, were synthesized as previously described [24]. Commercially available solvents and reagents were from Sigma-Aldrich (Milan, Italy) and used without further purification. Reactions and product mixtures were monitored by thin-layer chromatography on Merck F254 silica-gel-coated plates, using the indicated solvent systems. Flash chromatography was carried out with Merck silica gel (230–400 mesh). All drying operations were performed over anhydrous sodium sulfate and using a rotatory evaporator. Stock solutions of 10 mM in dimethyl sulfoxide (DMSO) were prepared and stored at −80 °C until their use.

### 2.2. Cell Cultures and Cytotoxicity Assay

TE85 (osteosarcoma), MG63 (osteosarcoma), TT (thyroid carcinoma), Colo38 (melanoma), SK-ChA-1 (cholangiocarcinoma), and Mz-ChA-1 (cholangiocarcinoma) cell lines were grown in DMEM with 10% (*v*/*v*) fetal bovine serum (FBS), streptomycin 100 μg/mL, and penicillin 100 U/mL. Cultures were maintained at 37 °C in a humidified incubator in an atmosphere containing 5% CO_2_.

In the cytotoxicity assay, adherent cells were detached by trypsin and seeded in a 96-well plate. After 8 h, the medium was replaced with fresh medium alone (control) or containing the examined compounds at various concentrations. After 72 h of growth, 100 μL of 0.5 mg/mL 3-(4,5-dimethylthiazol-2-yl)-2,5-diphenyl tetrazolium bromide (MTT) solution was added to the culture medium, and the cells were incubated for 4 h at 37 °C. An addition of 100 μL DMSO to each well allowed for the dissolution of formazan crystals. Absorbance measurement at 570 nm, using an Infinite 200 PRO multi-well plate reader (TECAN, Mannedorf, Switzerland), allowed for cellular evaluation. Alternatively, the optical count of cells in Burker’s chamber was performed. The IC50 value was estimated by using the Excel add-in ED50V10. Three independent experiments were performed in triplicate.

### 2.3. Clonogenic Assay

This assay aims to determine cell survival based on the ability of a single cell to grow and form a colony in the presence of the compound of interest. Osteosarcoma cells were plated in triplicate at 100 cells per well in six-well plates. The day after, the molecule under investigation was added to the culture medium, and the cells were cultured in DMEM containing 10% FBS for 21 days without renewal of the medium or the drug. After this time, the cells were washed with phosphate-buffered saline (PBS) and fixed for 10 min in absolute methanol. The colonies grown in a Petri dish were stained for 10 min with 0.5% crystal violet dye diluted in methanol and counted. Two independent experiments were performed in triplicate.

### 2.4. Cell Cycle Analysis and Determination of Apoptotic Cell Death

The cell cycle was investigated as previously described [25]. Briefly, after a 72 h treatment with the test substances, cells were harvested by trypsin and then fixed in cold 70% ethanol for 20 min. After washing in PBS, they were suspended in 0.2 mL PBS containing RNase A and propidium iodide (PI) for 30 min at 37 °C in the dark. The PI fluorescence of individual nuclei was measured by using flow cytometry. The percent of cells in the G0/G1, S, and G2/M phases of the cell cycle were automatically calculated by using the FlowJo software, version 9.9.6 (Tree Star, Ashland, OR, USA). The experiments were performed in triplicate and repeated three times.

Apoptosis was determined by using flow cytometry after Annexin V and Propidium Iodide (PI) dual staining. Cultured cells were incubated in 6-well plates in a medium containing IC50 concentration of the examined compound for 72 h. After this time, the control cells grown in a normal medium reached 70–80% confluence. The cells were harvested by trypsin, stained using an Annexin V/PI Kit (MabTag, Friesoythe, Germany) according to the manufacturer’s protocol, and immediately analyzed by flow cytometry, as previously described [25]. All samples were assayed in duplicate, and each experiment was performed three times.

### 2.5. Western Blot Analysis

Protein expression was investigated by Western blot, as described previously [26]. In brief, cells washed with PBS were disrupted in lysis buffer (Tris-Cl 50 mM, NaCl 150 mM, 0.02% NaN_3_, 1% Nonidet P-40, 0.1% SDS, 0.5% sodium deoxycholate, 5 μg/mL NaVO_3_, and 1 μg/mL leupeptin and aprotinin). After centrifugation, the recovered supernatants were subjected to a standard protein-quantification procedure. Equivalent amounts of protein for each sample were submitted to SDS–PAGE, and the gel was blotted to nitrocellulose membranes (Millipore, Bedford, MA, USA). After blocking with PBST (PBS containing 0.05% Tween 20) containing 5% nonfat milk for 1 h, each membrane was incubated with primary antibodies for 1 h at room temperature or overnight at 4 °C, and then the membranes were probed with peroxidase-conjugated secondary antibodies for 1 h at room temperature. The specific proteins were visualized by enhanced chemiluminescence.

### 2.6. UV–Visible Spectrum Analysis

The analysis of the UV–visible absorption spectrum of the molecules was performed in the range between 250 and 600 nm. CUR and its derivatives were diluted at 25 μM in 0.1 M phosphate buffer, pH 7.2, in the presence or absence of 2 µM albumin from bovine serum. The spectra were collected at 5 min intervals in the first 20 min of incubation and then at 40, 80, and 160 min.

### 2.7. Bioavailability Prediction Analysis

The comparison of the distinctive chemical–physical parameters of a potential drug with those of drugs already on the market allows us to predict its bioavailability in silico. Parameters such as molecular weight (≤500), presence of hydrogen bond donor groups (≤5), presence of hydrogen bond acceptor groups (≤10), and octanol/water partition coefficient (Log P, ≤5), were calculated as part of Lipinski’s five rules [27]. In addition, polar surface area (PSA, acceptable values ≤ 140 Å) [28] and molecular flexibility, based on the number of free rotatable bonds present in the structure (acceptable values ≤ 7) [29], were considered. Molecules whose parameter values do not exceed the limit should have good bioavailability. The Molinspiration Cheminformatics Software version 2014.11 performed the calculations of all the parameters.

### 2.8. Statistical Analysis

The results were expressed as the arithmetic mean ± standard deviation. Statistical calculations were performed by using a one-way ANOVA, and the differences among groups were examined by using the Bonferroni *t*-test. The *p*-values < 0.05 were significant.

## 3. Results

### 3.1. Design and Stability of the CUR-Derivatives in Solutions at Physiological pH

**Figure 1:**
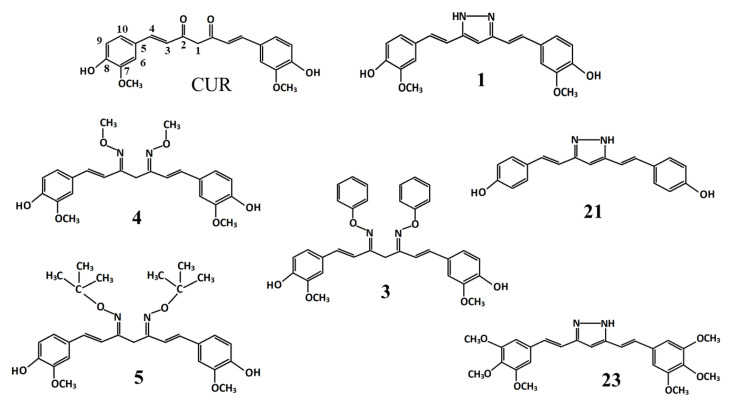
Chemical structures of curcumin and its derivatives.

In **1**, **21**, and **23**, the bis-ketone group was replaced with a pyrazole ring. In **1**, the substitutions on the benzene ring were the same as in CUR. In **21**, the methoxyl group in position 7 was eliminated as described above [30], and in **23** positions 7, 8, and 9 were occupied by methoxyl groups. Concerning the latter derivative, the absence of the hydroxyl group in position 8 should prevent its glucuronidation [31], thus potentially conferring better persistence in the blood. In derivatives **3**, **4**, and **5**, the bis-ketone group was modified into O-substituted oximes [24]. All compounds were dissolved in DMSO, and solutions ranging in color from pale yellow to colorless were obtained. The chemical structures of the derivatives are shown in Figure 1.

UV–visible spectrum analysis has been used to describe the instability of CUR molecule in slightly alkaline solutions at physiological pH and the stabilizing effects of albumin in phosphate buffer, in cell culture medium containing 10% FBS, and in human blood [10,19]. In a similar way, we studied the stability of derivatives.

**Figure 2:**
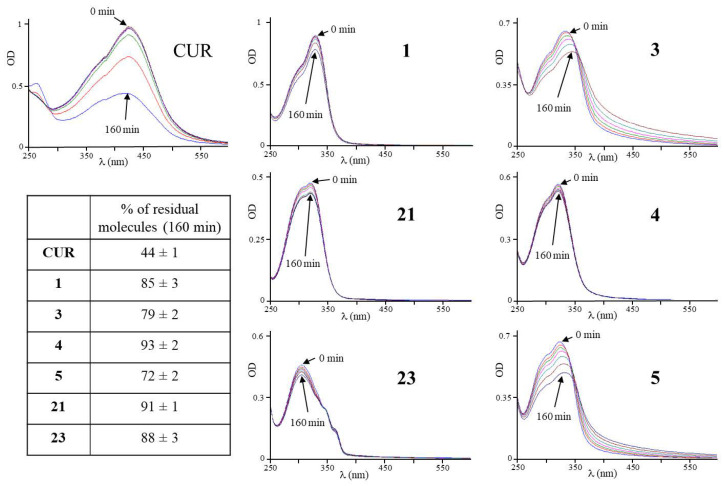
Time-dependent modification of the UV–visible spectrum of CUR and its derivatives in phosphate buffer, pH 7.2. Representative spectra are shown. Lower left inset: stability of derivatives. The residual percentage of molecules present after 160 min of incubation was estimated as the ratio between the absorbance value of the maximum absorption peak after 160 min and the value at time zero expressed as a percentage. The indicated values are the arithmetic means ± SD (*n* = 4).

In the first series of experiments, the stability of the derivatives at physiological pH 7.2 in phosphate buffer with respect to CUR was studied by comparing the spectra obtained at time 0; every 5 min up to 20 min; and then at 40, 80, and 160 min (Figure 2).

CUR had a maximum absorbance peak at approximately 420 nm, in the visible range, which decreased to 44% after 160 min. A new time-dependent peak appeared at about 260 nm, whose height was inversely related to the peak at 420 nm, and thus interpreted as an interconversion phenomenon consistent with the bis-ketone bond breaking and the formation of the byproducts of CUR already described [19]. Instead, the derivatives had a maximum absorption peak between 320 and 350 nm. The comparison between the spectra of the CUR and those of the derivatives allowed us to highlight the greater structural stability of all the derivatives in solution at physiological pH after 160 min. Some derivatives were particularly stable (**1**, 85%; **4**, 93%; **21**, 91; **23**, 88%), while others were less so (**3**, 79%; **5**, 72%), but still more stable than CUR (44%). Therefore, the replacement of the bis-ketone with a pyrazole ring or O-substituted oximes improved the stability of all the CUR-derivative molecules.

**Figure 3:**
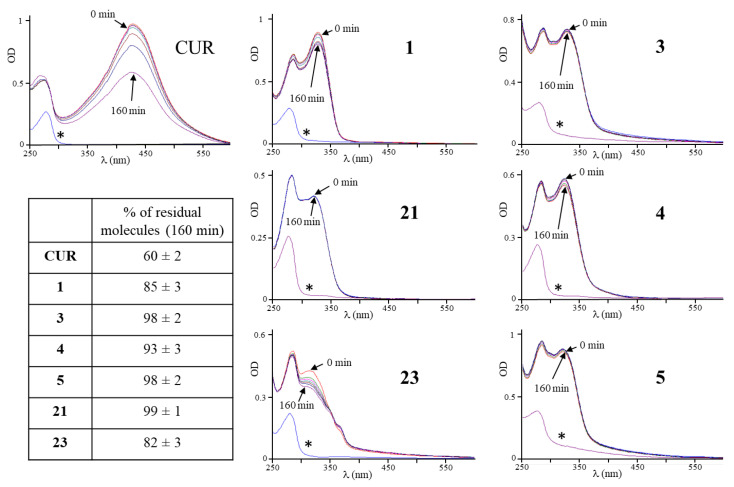
Time-dependent modification of the UV–visible spectrum of CUR and its derivatives in phosphate buffer, pH 7.2, containing albumin. Representative spectra are shown. Lower left inset: The stability of derivatives after 160 min of incubation was estimated as described in Figure 2. The indicated values are the arithmetic means ± SD (*n* = 4). The asterisk indicates the absorption spectrum obtained with the albumin-containing phosphate solution (blank).

Subsequently, a series of similar experiments conducted on derivatives in a phosphate buffer solution at physiological pH 7.2 and in the presence of albumin allowed us to study their stability (Figure 3).

In the presence of albumin, the stability of CUR increased to 60%, +16% compared to the value observed in the absence of albumin, which confirmed the instability of its structure within the analyzed times. The derivatives already stable in the absence of albumin confirmed their stability (**1**, 85%; **4**, 93%; **21**, 99%), except for **23** (decreased to 82%). The derivatives previously partially unstable in the absence of albumin further improved their stability in presence of albumin (**3**, 98%; **5**, 98%). Therefore, in albumin-containing biological fluids such as cell culture complete medium or human blood, derivatives might have higher stability than CUR.

### 3.2. Cytotoxicity of CUR and Its Derivatives on Cancer Cell Lines

The cytotoxicity of the CUR derivatives in cell lines stabilized from different types of cancer was analyzed. Cells were cultured in the absence or presence of increasing drug concentrations, and cytotoxicity was evaluated by MTT staining and expressed as the percentage of viable cells with respect to those present in the control well, treated with solvent alone (DMSO). Table 1 reports the drug concentration able to inhibit proliferation of 50% compared to untreated cells (IC50).

From the comparison of the IC50 values, CUR and its derivatives were cytotoxic in the panel of cancer cell lines tested, with IC50 concentrations ranging between 0.5 and 29.6 µM. Derivative **4** was always less active than CUR in all lines tested. Other derivatives were particularly more active than CUR in certain cancer cell lines: **1** and **3** in melanoma, thyroid cancer, and OS cell lines; **5** in melanoma and thyroid cell lines; **21** in OS cell lines; and **23** in the thyroid cancer cell line. The different cytotoxic effects produced by each derivative in the different cell lines—sometimes enhanced and sometimes weakened—with respect to the CUR, underlined how cytotoxicity was related not only to the modifications introduced in the molecule but also to the cell type.

**Figure 4:**
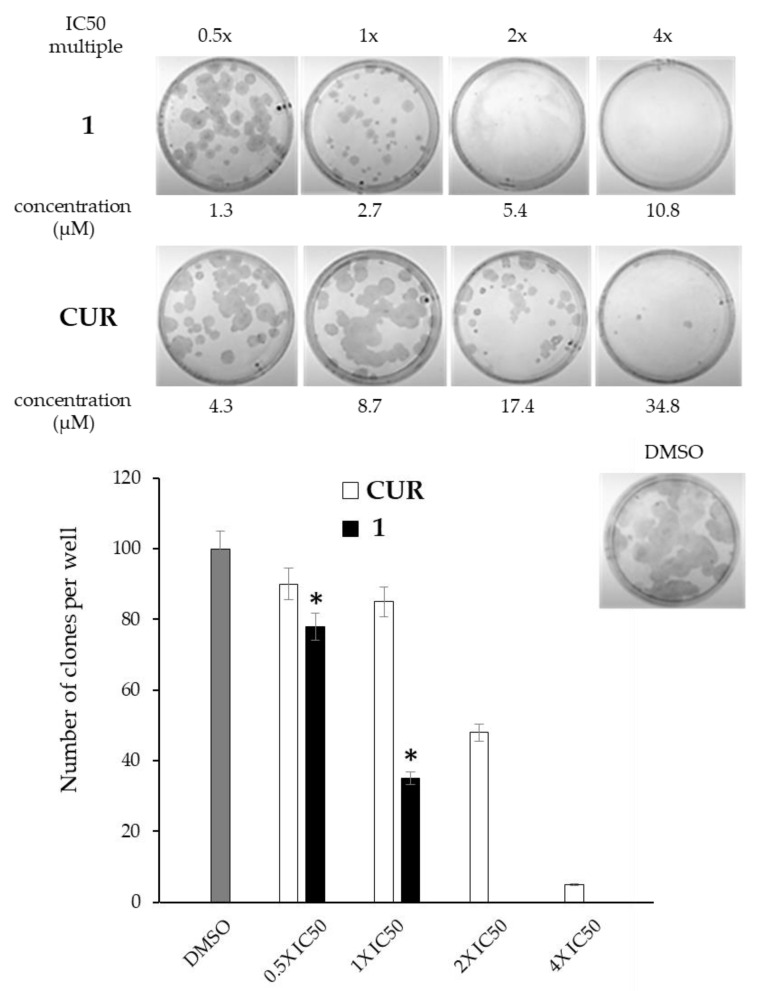
Inhibition of the clonogenic activity of MG63 in the presence of increasing concentrations of **1** or CUR. Each compound was tested at drug concentrations equivalent to multiple (2×, 4×) or fraction (0.5×) of the IC50 concentration (1×). Upper panel: representative view of clones grown after 21-days of administration of **1**, CUR, or DMSO. Lower panel: statistical representation of two experiments performed in triplicates. Arithmetic means ± SD were reported in the histogram. The asterisks indicate the significant difference in the values found for the derivative with respect to the CUR.

Derivative **1** was the most active among the others, showing accentuated cytotoxicity in OS cell lines MG63 (2.7 μM) and TE85 (0.5 μM) with respect to CUR (8.7 μM and 9.5 μM, respectively). It is noteworthy that the concentrations at which **1** had a marked cytotoxicity on OS cells were very close to the CUR plasma value obtained in a clinical study [9] and deserve further investigation. The greater stability in physiological pH solutions of **1** with respect to CUR might allow a longer duration of its cytotoxic effect. Therefore, the long-lasting cytotoxicity of **1** with respect to CUR in MG63 cells was investigated by the clonogenic assay (Figure 4)

The comparison of the long-lasting effects of **1** versus CUR was performed by administering to MG63 cells adherent on the plate concentrations comparable in terms of pharmacological efficacy (IC50, fraction of, and multiples), and the number of clones was counted after 21 days. Derivative **1** and CUR dose-dependently reduced the ability to generate cell clones but exhibited different behavior. In fact, only by using the CUR in a double concentration of the IC50 value was it possible to obtain a number of clones comparable to the number present in the plate treated with **1** at the IC50 concentration (35 ± 5). The previously observed higher stability of **1** in biological fluids than CUR could explain the latter finding. In addition, CUR at cytotoxic concentrations (2× and 4× the IC50 value) did not show a total absence of clones in the plate, while this condition was achieved with equivalent doses of **1**. These observations have shown how the cytotoxicity of **1** was maintained even in conditions of long treatment, presumably by virtue of its greater stability in biological fluids.

### 3.3. Effects of the Derivatives on the Cell Cycle and Induction of Apoptosis in OS Cells

**Figure 5:**
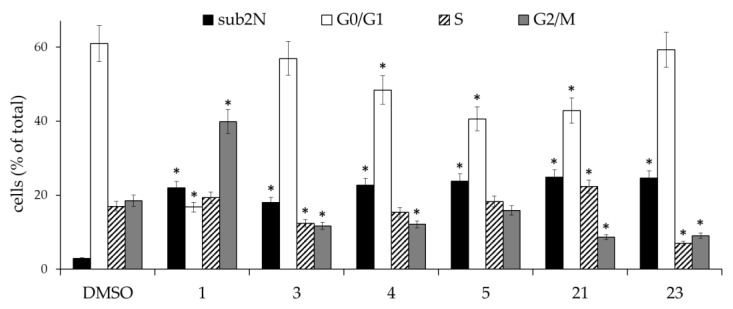
Effects of the derivatives on the cell cycle in MG63 cells. The cells were treated for 3 days in the presence of derivative at the IC50 concentration, stained with PI, and processed by flow cytometry. The values are expressed as arithmetic mean ± SD (*n* = 6); sub2N, sub-diploid peak. The asterisk indicates a significant difference (*p* < 0.05) between the value found for a specific phase of the cycle following treatment with the derivative and the value obtained for the same phase following treatment with DMSO.

Therefore, we evaluated the effects of the derivatives on the cell cycle and on the induction of apoptosis. For this purpose, OS MG63 cells were cultured for 72 h in the presence of each derivative at the IC50 concentration or DMSO as a reference. Figure 5 reports the effects on the cell cycle observed by flow cytometry.

**Figure 6:**
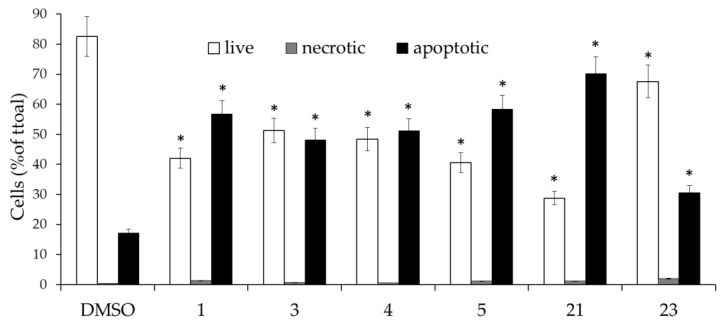
Effects of the derivatives on the cell death of MG63 cells. The cells were treated for 3 days in the presence of the derivative at the IC50 concentration, stained with Annexin V/PI, and processed by flow cytometry. The values are expressed as arithmetic mean ± SD (*n* = 6). The asterisk indicates a significant difference (*p* < 0.05) between the value found for a specific population following treatment with the derivative and the value obtained for the same population after the treatment with DMSO.

All derivatives were able to modify the cell cycle of OS cells, with slightly different effects. All of them caused a significant increase in the sub-diploid peak (sub2N); therefore, they led us to suppose they were capable of triggering apoptosis. Furthermore, on another aliquot of each sample, the staining with Annexin V/PI permitted the analysis of apoptosis (Figure 6).

The results confirmed the ability of the derivatives to induce apoptosis in OS cells, as is associated with a low level of necrosis, two important characteristics for a potential anticancer drug.

**Figure 7:**
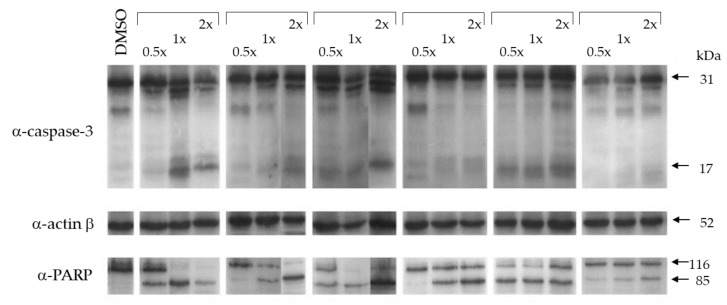
Western blot analysis of the caspase-dependent apoptosis. The MG63 cells were treated with pharmacologically equivalent concentrations of the derivatives or DMSO: 0.5× = half the IC50 concentration, 1× = IC50 concentration, and 2× = double of the IC50 concentration. The uncropped blots are shown in Figure S1.

Furthermore, the Western blotting experiment shown in Figure 7 allowed us to verify if the derivatives induced apoptosis through the activation of a caspase-dependent mechanism. MG63 cells were cultured for 72 h in the presence of increasing concentrations of derivative (multiples or submultiples of the IC50) or DMSO and then subjected to analysis, using specific antibodies, which evidenced the structural changes responsible for caspase 3 activation and the related PARP inactivation.

The levels of pro-caspase-3 (31 kDa) decrease following the triggering of apoptosis, due to the proteolytic cleavage that releases the active caspase 3 (17 kDa), whose levels increase. Furthermore, activated caspase 3 performs a proteolytic cut on the PARP protein (116 kDa), transforming it into the inactive form (85 kDa).

All derivatives, except for **23**, showed a significant increase in active caspase 3 (17 kDa) in the range of the concentrations analyzed. Conversion of PARP from the active (116 kDa) to the inactive (85 kDa) form confirmed the activation of caspase 3. Concerning compound **23**, although the conversion of procaspase to caspase 3 was not visible by using the α-caspase 3 antibody, our analysis of the conversion of PARP suggested that it was able to activate apoptosis through a caspase3-dependent mechanism, but only at very high doses. In this respect, derivative **23** showed levels of apoptosis equivalent to those of the other derivatives in the analysis of the sub-diploid peak (Figure 5), while it showed lower activity in the analysis of a caspase-dependent mechanism such as the extraversion of phosphatidylserine (Figure 6). Therefore, it is reasonable to hypothesize that this compound could trigger not only the caspase3-dependent mechanism but also other pathways of apoptosis activation.

From these results, it emerged that all derivatives were able to trigger the apoptosis of OS cancer cells through a caspase-3-dependent mechanism.

### 3.4. Prediction of Oral Bioavailability of Curcumin Derivatives

To predict whether derivatives might have drug-like properties characteristic of proven good drugs, we used the Molinspiration Cheminformatics Software. Table 2 reports the values obtained for the different parameters. Molecules exhibiting values within the limits presumably should have good bioavailability.

The CUR, which has a known limited bioavailability, respected Lipinski’s rule but exceeded the value of the number of rotating bonds, which were eight against a maximum of seven. With regard to derivatives, some of them may exhibit lower bioavailability with respect to the lead CUR molecule. In fact, **3** and **5** have shown two violations of Lipinski’s rule, one was logP or molecular weight, and another violation was the number of rotatable bonds. Derivatives **4** and **23** respected Lipinski’s rule but exceeded the limit of the number of rotating bonds like the CUR. Therefore, compared to the latter, these derivatives did not represent an improvement in terms of predicted bioavailability. Notably, **1** and **21** complied to all parameter limits and, therefore, should have better characteristics with respect to CUR, and, thus, they should permit higher blood levels to be reached than those observed in the CUR trials.

## 4. Discussion

The results showed that the replacement of the bis-ketone moiety of the CUR significantly improved the stability of the derivatives in solutions at physiological pH, both in the absence and in the presence of albumin. In particular, as regards the substitution of the bis-ketone with two oximes introduced in the **3**, **4**, and **5**, the spatial size of the groups bound to the oxime seemed to contribute in an inversely proportional way to the stability of the molecule in biological fluids. In fact, the **4** characterized by the presence of a methyl group linked to the oxime was stable even without the contribution of albumin. The derivatives having very large groups linked to the oxime (**3**, phenyl; **5**, *tert*-butyl) were less stable than **4**. However, in the presence of albumin, all derivatives containing the oximes were very stable. Instead, as regards **1**, **21**, and **23**, the pyrazole ring conferred comparable stability to the molecules, always greater than the CUR in solution at physiological pH regardless of the substituents introduced into the aromatic rings. Therefore, the simple replacement of the bis-ketone with a pyrazole ring was sufficient to stabilize the derivative in solutions at physiological pH.

From the point of view of their cytotoxic activity, the increase in stability did not necessarily translate into molecules that are more active. In fact, despite the great stability observed for **4**, the molecule was less active than the CUR in all the cancer cell lines tested. From this observation, it was possible to conclude that the molecular determinants that influence stability were not automatically the same ones that influenced the cytotoxicity of the derivative. In particular, for derivatives containing oximes, large substituents (phenyl or *tert*-butyl) linked to the oxime appear to enhance the cytotoxicity of the molecule, as observed for **3** and **5**, compared to small groups such as the methyl present in **4**. As far as the pyrazole derivatives are concerned, the replacement of the bis-ketone group of the CUR with a pyrazole was not a sufficient condition to obtain a derivative with higher cytotoxicity. In fact, the pyrazole derivative **1**, which had a structure very close to that of CUR, was as active as the latter in cholangiocarcinoma lines. Of course, the replacement of the bis-ketone with pyrazole seemed to give the molecules a certain degree of specificity for OS. In fact, both **1** and **21** expressed their greatest cytotoxicity on OS lines compared to the other cancer cell lines. From the observation of the cytotoxicity profile of **23**, the type and number of substituents bound to the aromatic rings also seemed to orient its cancer-specific activity, since this molecule showed a marked cytotoxicity in thyroid cancer cells compared to the other lines tested. Furthermore, from the comparison of structures **1** and **21**, it emerged that the concomitant presence of the 7-methoxyl and 8-hydroxyl groups in the aromatic rings of **1** conferred a higher cytotoxic potential. Indeed, elimination of the 7-methoxyl group in 21, or the substitution of the 8-hydroxyl with a methoxyl group in **23**, produced less active molecules.

Derivative **1** aroused particular interest since it was the most active in terms of cytotoxicity among all others studied. In particular, in the OS cell lines, its IC50 value was comparable to the plasmatic concentration of CUR observed in patients in clinical trials. This derivative has not only been shown to possess a stronger short-term (three-days cytotoxicity assay) but also long-term (21-days clonogenic assay) anti-proliferative activity with respect to CUR. Therefore, from a therapeutic perspective, it could exert pharmacological effects of longer duration than the latter. Furthermore, along with the other derivatives tested on OS cells, **1** triggered minimal necrosis, an optimal requirement for a potential drug to be used in human therapy, as well as the ability to modify the cell cycle of OS cells by activating the caspase cascade and the consequent cell death by apoptosis.

## 5. Conclusions

The better chemical–physical characteristics of **1** with respect to CUR allowed for predicting a better future bioavailability. The greater molecular stability of **1** in biological fluids and its higher cytotoxicity on OS cells, with respect to CUR, even for a long period after administration, were the strength points of this derivative. In perspective, this interesting derivative **1** would deserve to be studied in future clinical trials on the treatment of OS, either alone or in combination with other drugs currently in use.

## Figures and Tables

**Table 1:** Cytotoxicity of CUR and its derivatives on cancer cell lines growing in adhesion. Viable cells were evaluated 72 h after the treatment with increasing concentrations of the indicated molecule. The values indicate the IC50 concentration (μM) ± SD. Values in red indicate a greater cytotoxic effect than CUR.

	Colo38	TT	SK-ChA-1	Mz-ChA-1	MG63	TE85
**CUR**	13.6 ± 0.7	13.5 ± 0.9	4.6 ± 0.3	7.6 ± 0.6	8.7 ± 0.7	9.5 ± 0.7
**1**	6.3 ± 0.4	6.0 ± 0.5	8.2 ± 0.3	8.8 ± 0.3	2.7 ± 0.2	0.5 ± 0.03
**3**	8.7 ± 0.6	9.1 ± 0.7	10.9 ± 0.9	12.4 ± 1.0	11.8 ± 0.7	7.6 ± 0.4
**4**	20.2 ± 1.6	16.7 ± 1.3	21.1 ± 1.1	16.7 ± 1.1	23.5 ± 1.2	16.7 ± 1
**5**	10.3 ± 1.8	7.2 ± 0.4	8.2 ± 0.5	6.6 ± 0.5	8.8 ± 0.7	9.5 ± 0.8
**21**	29.6 ± 1.7	15.8 ± 0.9	27.9 ± 2.2	28.3 ± 1.4	26.3 ± 1.3	6.9 ± 0.4
**23**	15.4 ± 0.9	10.6 ± 0.5	24.2 ± 1.4	18.7 ± 1.1	28.2 ± 1.7	20.2 ± 1.5

**Table 2:** Molecular physicochemical properties of CUR derivatives. LogP = octanol/water partition; MW = molecular weight; nON = number of hydrogen bond acceptors; nOHNH = number of hydrogen bond donors; nrotb = number of rotatable bonds; PSA = polar surface area. Values that exceed the limit are indicated in red.

Limit Values		CUR	1	3	4	5	21	23
≤5	**LogP**	2.3	4.23	**7.15**	3.75	**6.12**	4.60	4.82
≤500	**MW**	368.38	364.40	**550.61**	426.47	**510.63**	304.35	452.51
≤10	**nON**	6	6	8	8	8	4	8
≤5	**nOHnH**	2	3	2	2	2	3	1
≤7	**nrotb**	**8**	6	**12**	**10**	**12**	4	**10**
<140 Å	**PSA (Å)**	93.07	87.61	102.12	102.12	102.12	69.14	84.09
≤1	**violations**	1	0	**3**	1	**3**	0	1

## Data Availability

Not applicable.

## Supplementary Materials

The following supporting information can be downloaded at: https://www.mdpi.com/article/10.3390/life13020431/s1, Figure S1: The uncropped blots.
