# Supplementary figures and images for: Characterization of Stable Pyrazole Derivatives of Curcumin with Improved Cytotoxicity on Osteosarcoma Cell Lines
Source: Life (Basel). 2023 Feb 3;13(2):431. doi: 10.3390/life13020431 (PMC9961829; doi:10.3390/life13020431)

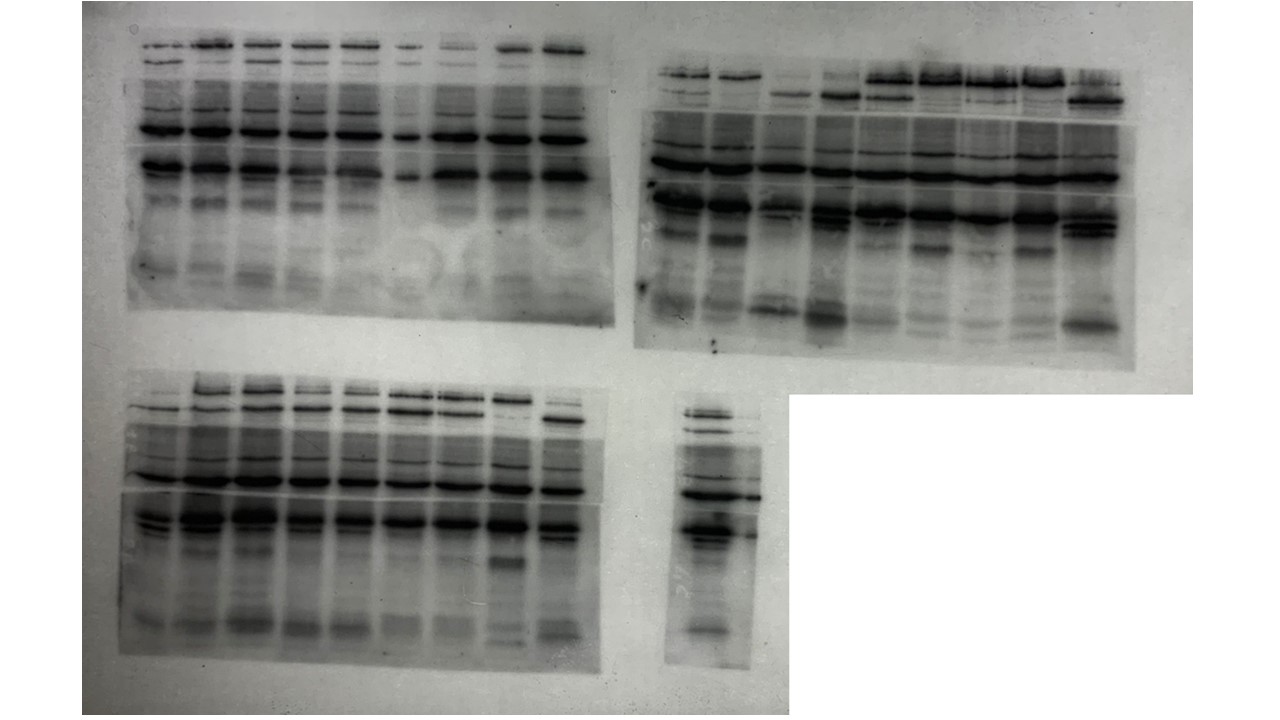

Supplement: Supplementary file 1 [file life-13-00431-s001.zip › life-2131652-supplementary.jpg]
